# Lion trophy hunting in West Africa: A response to Bouché et al.

**DOI:** 10.1371/journal.pone.0173691

**Published:** 2017-03-21

**Authors:** H. Bauer, P. Henschel, C. Packer, C. Sillero-Zubiri, B. Chardonnet, E. A. Sogbohossou, H. H. De Iongh, D. W. Macdonald

**Affiliations:** 1 Wildlife Conservation Research Unit, Zoology, University of Oxford, Recanati-Kaplan Centre, Tubney, United Kingdom; 2 Panthera, New York, New York, United States of America; 3 Department of Ecology, Evolution and Behavior, University of Minnesota, St. Paul, Minnesota, United States of America; 4 African Protected Areas & Wildlife, Saint Cloud, France; 5 Laboratory of Applied Ecology, University of Abomey-Calavi, Cotonou, Benin; 6 Institute for Environmental Sciences, University of Leiden, Leiden, The Netherlands; U.S. Geological Survey, UNITED STATES

Bouché et al. [1] recommend the continued reliance on sport hunting of African lions (*Panthera leo*) for the conservation of the W-Arly-Pendjari Protected Area complex (WAP). However, their survey techniques are inappropriate for providing precise estimates of lion population size at the scale used in their model, and their suggested quotas are excessive; consequently, their conclusions are unsupported.

Bouché et al. present lion numbers based on lion spoor (pugmarks) found while driving on unpaved roads and converted to lion numbers using a widely adopted methodology [2]. However, [2] demonstrated that calculations based on fewer than 30 separate spoor yield unreliable results (Coefficient of Variation (CV)>20%), so surveys should be designed to attain this minimum. We combined data from their S2 Table, with information from their report at http://aires-protegees.uemoa.int/sites/default/files/inv_carnivore_arly_pendjari2014.pdf to calculate CVs (our S1 Table). Although their 2014 survey provided robust estimates across the entire WAP complex (based on 97 independent spoor records), their research effort was not adequate for separate consideration of each of the 16 Hunting Zones (HZs). Our S1 Table shows a mean total transect length of 45.2km and a mean of 2.9 spoor per HZ. Only one HZ (with 12 spoor) had a CV of 41%, in the remaining HZs CVs varied from 94–141%, with an average of 105%. Nine HZs had 0 or 1 spoor, thus a CV could not be calculated, but if we use the mean value of 105%, we would find an estimated range of 18–414 lions in all HZs combined. The estimates we recalculated based on the raw data are higher due to two discrepancies; (1) the original survey report lists Porga and Batia as two separate HZs with a total area of 1823 km^2^, but Bouché et al. list them as one HZ with an area of 1513 km^2^, and (2) we were unable to reproduce the stratification based on distance to water. However, out critique is not about accuracy of estimates at ecosystem level for which we accept the figures reported by Bouché et al., the point here is rather the precision at HZ level. The confidence interval for almost every separate HZ includes a value of zero, thus it is impossible to make inferences about HZ-specific sustainable hunting quotas from these data. The data requirements for the analyses attempted by Bouché et al. are unlikely to be obtained through spoor transects, but we can use this method and the available data for an alternative analysis, leading to different conclusions about adequate quotas for WAP, which we will set out below.

In a parallel argument, Bouché et al. claim that any lion footprint over 12cm long represents a large adult male, and estimated a total of 168 large adult males (equivalent to 40% of the population of lions over 1 year old). They then suggest that a quota of 10 large adult males would fall safely below the recommended offtake of 10% of males as recommended by [3]. However, the precise relationship between age/sex and spoor size is unknown, and their result suggests a demographic profile for the WAP population that has never been reported elsewhere–uniqueness that, at the least, raises caution over its validity. For comparison, the proportion of adult males in other populations, excluding small cubs, was 16% in Etosha, Namibia [4] and 22% in Maasai Mara, Kenya [5]. Data from the long-term Serengeti lion study, show that males above 6 yrs of age (the recommended age-minimum for undisturbed populations [6]) typically comprise only 5–15% of the population (excluding small cubs), and males above 8 yrs of age (the recommended age minimum for disturbed populations [7]) typically comprise only 4–6% (Fig 1a). Indeed, in nearly 50 yrs of research, these proportions never exceeded 30% for 6-yr old males or 18% for 8 yr olds (Fig 1b). The only plausible way that the WAP population approached 40% would have been if the larger tracks included males that were as young as 2 yrs of age–far too young to be acceptable trophies.

**Fig 1:**
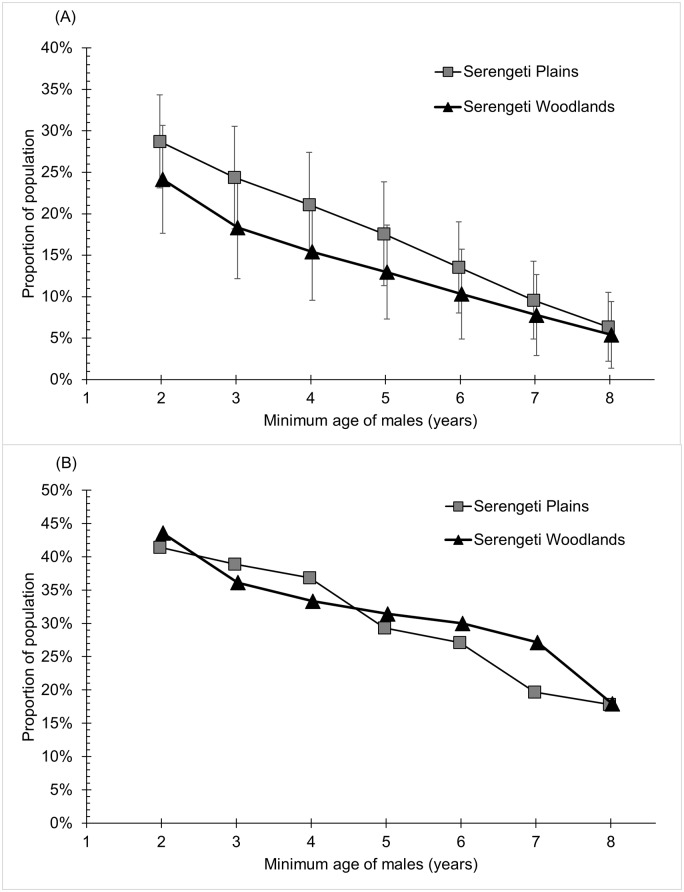
Number of males above each respective age divided by the total number of individuals of at least 1 yr of age in that population. Proportions were calculated for each month from 1966 to 2014 (582 mos in the Serengeti woodlands, 515 mos in the Serengeti plains, see [22]). (A) Average monthly proportions, vertical bars indicate standard deviations. (B) Maximum monthly proportion observed across the entire study period.

Secondly, by taking a percentage of the entire population, Bouché et al. are including lions from the NPs in the quota for the HZs through the so-called vacuum effect [3]). Lion hunting in adjacent HZs has previously been shown to have adverse effects on lion sub-populations in the National Parks of WAP [8] and elsewhere [3, 9]. It is likely that source-sink movements contributed to the sustained high harvests in WAP but this is not explored by Bouché et al.

The recommended harvest rate of 0.5 lions per 1,000km^2^ is widely adopted but was calculated for areas with lion densities of *ca*. 5 lions per 100km^2^ [9], recent evidence suggests that this rate is not appropriate for lower densities as shown by the very high extinction probability in an area with two lions per 100km²[7]. Despite WAP having only 1.6 lions per 100 km^2^, Bouché et al. (their Figs 2 and 5) show that harvest rates exceeded 0.5 lions per 1,000km^2^ in almost all HZs in all years. Both the lion hunting quota and harvest in Burkina Faso are the least cautious in Africa, as measured by various parameters [10]; in their S1 Table Bouché et al. reported an average quota of 4.2 and harvest of 2.0 lions per 1,000km^2^. Bouché et al. have tried to argue that harvests have been sustainable but nevertheless recommend a lower quota of 10 lions, corresponding to 1 lion per 1,000km^2^, which is recommended only for the high density population in Selous[9]. We have argued above that the data do not support their findings. Past off-takes may only have been sustainable as lions were drawn in from adjoining national parks through the so-called vacuum effect [3].

Moving away from the analysis at HZ level, we can derive alternative quota using their estimate of 190 lions in HZs plus 228 lions in NPs. One method is to use [9] adapted for the local lion density, thus 0.16 lions per 1,000km^2^, giving a quota of 1.6 lions per year, more practically formulated as 3 lions in two years. An age based approach based on the review above with ~10% of the male population above 6 yrs would give 19 individuals, giving a quota of 2 based on[3]. Yet another approach is to take 2.7–4.3% of the adult male population[11], approximately 1–2 lions. Quota were recently reduced to 11 (5 in Benin, 6 in Burkina Faso; Unpubl.), while this is an improvement quota for all HZs combined (Benin and Burkina Faso) should not exceed 2 lions per year, using internationally recommended quota-setting approaches.

It is important to introduce a proper quota, but there is a much bigger issue with the claim of Bouché et al. that management of HZs may collapse if lion trophies cannot be exported. Whichever quota is used, income for the hunting outfitter at current market price is only around 15,000 USD per lion and its economic impact, including any increased price for non-lion trophies, is unlikely to provide significant benefits. Lion hunting packages and the trophy fees which go to wildlife authorities (only 1600 USD in Burkina Faso; http://www.safari-evasion.com/burkina_evasion/chasse/taxes_abattage.php) are still by far the lowest on the continent in Benin and Burkina Faso, even though West Africa has by far the rarest lions, belonging to a distinct sub-species[12], and listed as Critically Endangered[13]. We doubt that hunting will remain a viable management model in the long term, not based on normative but on economic arguments. Continuing to kill Africa’s rarest lions for such perversely low revenues represents a market failure and will not further lion conservation. Lion persistence in WAP is most strongly linked to the number of patrol staff and average annual management budgets per km^2^, and minimum operations budgets for site protection of 125 USD/km2 (excluding ranger salaries) are needed to assure lion persistence[14]. This translates to an absolute minimum of 1.3 million USD (excluding ranger salaries) for all HZs.

The trophy hunting model collapsed in Central African Republic [15], is currently disintegrating in Cameroon [16], and could soon fail in WAP owing to the pervasive political insecurity in the region, and the low revenues achieved[17]. Revenues in WAP only cover a fraction of total management costs[17], which is also observed in other areas. Prior to 2012, net profitability of trophy hunting was marginal in Zambia and Namibia and negative in Mozambique. In Tanzania net profit was 158 USD/km²/year, but management costs have since risen from ~200 USD/km2/year [18] to an average of $830 ± $285/km^2^/year, partly in response to human population growth and a surge in poaching [19]. At present, 31% of Tanzania’s hunting blocks are unleased (unpubl.), and 40% of Zambia HZ area is encroached by local communities [20].

Overall, trophy hunting may have contributed to the persistence of the Critically Endangered lion population in WAP to some degree. But the situation is precarious, making it particularly important that recommendations are based on the highest quality data. We doubt that trophy hunting will make a meaningful contribution, unless it is limited to 2 lions annually auctioned to the highest bidder, as is the case with black rhinoceros in Namibia, with some individuals fetching close to 400,000 USD. In the face of the enormous challenge to find long-term funding for large tracts of Protected Areas across Africa [19, 21], new approaches to wildlife conservation are urgently needed.

## Supporting information

S1 Table Lion survey results in the WAP. Columns 1–5 are taken from Bouché et al. [1] and columns 6–7 are from their original survey report, the other columns show our original calculations. Note that distance to water is not a significant factor at the level of individual Hunting Zones and therefore we calculated estimates without stratification, which explains the difference in the total.
(XLSX)
